# Continuation of Mrs. Thomas's Case of Abscess in the Liver, with Dissection
*Vide Med. and Phys. Journal, No. 179, vol. xxxi, page 8.


**Published:** 1814-11

**Authors:** Robert Semple

**Affiliations:** Member of the Royal College of Surgeons in London. 35, High-street, Islington


*For the Medical and Physical Journal.*

### *Continuation of Mrs. Thomas's Case of Abscess in the Liver, with Dissection;** by Mr. R. Semple.

MAY 13th, 1814.—Her abdomen appears larger than before the last operation, with evident fluctuation. Fæces black and offensive. She had a dose of salts two days ago, which procured eight loose motions. Acute pain in the right side and shoulder. No stool to-day. Makes water freely, and in abundance. Habeat, Sulphat Sodæ ℥i. cras mane.

15th.—This day I proceeded to the operation as before. On introducing the trocar and canula, a discharge of pus, mixed with a little blood, took place, but the canula soon was stopped up, and I was obliged to withdraw the instrument, after having evacuated a few ounces only of the matter. Quiescat, et Habeat, Opii gr. iss.

16th.—Feels better. No pain in the wound. Passed a good night. Bowels open. Stools dark. Repet. Opium, horâ somni.

18th.—Habeat, Sulphat Sodæ ℥j. statim.

22d.—At eleven o'clock this morning, another surgeon of

* Vide Med. and Phys. Journal, No. 179, vol. xxxi, page 8.

this this town accompanied me to the work-house, when it was determined to perform the operation as before. I therefore punctured the abdomen in the linea alba, mid-way between the pubes and umbilicus, with a large-sized canula and trocar, and evacuated by measure upwards of two gallons of good conditioned pus, free from smell. During the operation, several gelatinous bodies similar to hydatids obstructed the passage of the matter through the canula. The probe and forceps soon removed these substances, and the matter flowed copiously. The patient bore the operation well, and complained of no particular uneasiness, save when pressure was applied to the region of the liver. A slight hæmorrhage ensued when the canula was withdrawn: perhaps some small branch of an artery might have been wounded, and did not shew itself, till the pressure of the canula was removed. It, however, soon stopped. The health, pulse, and spirits, have, since the last operation, been good. Indeed no function, but the liver's secretion, seemed in any way to be depraved. Her bowels have been seldom moved, without the aid of laxatives. After the operation, I ordered the following medicine:

℞. Tinct. Opii M. xl.

Aq. Menth. Pip. ℥ifs. M. fiat haustus statim sumendus.

At five o'clock in the evening I saw her. She appeared cheerful, and, upon the whole, free from pain. She had eaten some lamb and spinage with relish. Her countenance was expressive of much satisfaction at the success of the operation. The assistance I experienced from Mr. William Hole, the medical gentleman above alluded to, was of material service to me in the performance of the operation. I ordered her draught to be repeated at eight o'clock in the evening, with thirty minims of the Tinctura Opii.

As I have published part of this case already in the Medical and Physical Journal, I hold it right to make the result of it known. It is an interesting and important case. It was at one time suggested to me, that the matter might have been contained in the sheath of the rectus muscle; but its being diffused all over the abdomen, its quantity, &c. removed entirely that supposition.

May 23d.—Passed a comfortable night. No pain in the wound. Tongue clean. Pulse good. Bowels rather confined. Hab. Sulphat Sodæ ℥j. statim.

24th.—Salts operated. Feels comfortable, and is better than I could have expected. She has every kind of nourishment, with wine and porter. No pain in the wound. Quiescat.

29th.—Symptoms of hysteria.

℞. Gum. Ferulæ Assæ-Fœtidæ gr. xv. Divide in pilulas duas; statim sumendas.

 31st.

31st.—Removed the plaister, and found the wound healed up. Voided much urine this morning. Belly soft, and breathing free. Tongue clean. Bowels confined. Sits up, and eats well. Sumat omni nocte Pilulæ Hydrargyri gr. vj.

June 12th.—Bowels very loose, and the motions mixed with scybalæ, and tinged with blood. Slight ptyalism. Habeat Extracti Opii gr. ij. h. s.

16th.—Mouth sore. Slept well. Rep. Opium. Capiat Pilulæ Hydrargyri gr. iij. 4ta quaque nocte tantum.

24th.—Mouth well.—Has had the opium since last report. Hab. Sulph. Sodæ ℥ifs. statim. Omittantur alia medicamenta.

25th.—Salts procured about twelve motions, slimy, scybalous, and mixed with blood.

26th.—Has had a great number of bilious stools since last report. Feels easier. Belly soft. Habeat Extracti Opii gr. ifs. horâ somni.

27th.—Says she feels great relief from the opium, urgently wishing it to be continued. Purging stopt. Makes abundance of urine. Rep. Opium.

July 2d.—Teeth loose. Ptyalism inconsiderable. Says her stomach rejects all ingesta, and is only allayed by the opium. Repet. Opium.

14th.—Her breasts, which were formerly flaccid, have this morning swelled. Belly enlarging apace. Mouth well. Continuetur opium.

22d.—Legs swollen, erysipelatous, and painful.

℞. Superacetat. Plumb. ℈j.
Aquæ Puræ ℔ij. Solve. Humectentur crura frequenter more solito. Rep. Opium.

August 11th.—A small pustular tumour is observed on the cicatrix where the last operation was performed. Continuentur Medicamenta.

12th.—The small tumour burst, and about an ounce of pus was discharged. It does not appear to communicate with the abdominal cavity. Continuentur Medicam. Applicetur Emplastrum Adhæsivum parti.

18th.—Hysteria.

℞. Gum. Assæ-Fœtid. ℈fs. divide in pilulas duas, statim sumendas.

19th.—Says she has been very ill during the night. The place where the puncture was made, last operation, discharges a small quantity of pus. Hab. Extr. Opii gr. ij. horâ somni.

22d.—Passed two large potsful of thin matter, which she could could not well describe, and the nurse had emptied the vessels. Belly softer. Augeatur dosis Opii ad gr. iifs.

23d.—The quantity of watery fetid matter which she passes per anum is incredible. Has changed in her countenance for the worse. Repetatur Opium.

September 17th.—She has gradually sunk, and her faculties have remained entire, and she this afternoon, without a struggle, expired.

*Dissection.*—September 19th, 1814.—I first drew off with the canula and trocar about a gallon and a half of good conditioned pus, and then divided the parietes of the abdomen in the usual manner. The first thing that presented itself to me was a general blush of inflammation on the surface of the abdominal viscera; and on pulling aside the flaps of the abdominal parietes, a considerable quantity of the same kind of matter which I had drawn off by the instrument appeared. This I sponged clean out, and discovered an immense cavity filling the chief part of the hypogastric region, reaching as high as the diaphragm, and lying immediately under the liver. This last viscus, together with the gall bladder, were to all appearance sound and healthy, and on cutting into the substance of the liver, it was found free from disease. The bag of the tumour did not communicate with any viscus of the abdomen. Under the bag of the tumour lay the uterus and bladder, in a sound and healthy state. The internal surface of the sac or bag of the tumour was interspersed with several black spots, and a hydatid of a very enormous size was found slightly attached to its lower part. The sac adhered firmly to the circumjacent parts; it was about the fifth of an inch in thickness, and of a capacity to contain about three gallons. It extended from ilium to ilium. The ovaria and fallopian tubes were to all appearance sound. Except this bag of purulent matter, and the inflammatory appearance already described, no other symptoms of disease were discernible in the abdominal cavity. As this woman had never complained of any pain or uneasiness in the thorax, that cavity was not opened.

My idea of this disease being an abscess in the liver, was, perhaps, a justifiable one, from the tumour lying immediately under that viscus. The cavity of this abscess having no communication with any of the viscera, and its immediate connection and adhesion to the integuments of the abdomen, renders it extremely possible that a permanent opening externally might have been beneficial. I proposed the operation some time previous to her decease, but she objected to it, and delayed her compliance till it was too late.

I may

I may here remark, that so prevalent was the supposition that this woman was with child, that my assistant was asked by the porter's wife at the work-house lodge, after the dissection, whether this circumstance was not true.

ROBERT SEMPLE,
*Member of the Royal College of Surgeons in London.*

35, *High-street, Islington;*

